# Cascade surface modification of colloidal quantum dot inks enables efficient bulk homojunction photovoltaics

**DOI:** 10.1038/s41467-019-13437-2

**Published:** 2020-01-03

**Authors:** Min-Jae Choi, F. Pelayo García de Arquer, Andrew H. Proppe, Ali Seifitokaldani, Jongmin Choi, Junghwan Kim, Se-Woong Baek, Mengxia Liu, Bin Sun, Margherita Biondi, Benjamin Scheffel, Grant Walters, Dae-Hyun Nam, Jea Woong Jo, Olivier Ouellette, Oleksandr Voznyy, Sjoerd Hoogland, Shana O. Kelley, Yeon Sik Jung, Edward. H. Sargent

**Affiliations:** 1 Department of Electrical and Computer Engineering, University of Toronto, 10 King’s College Road, Toronto, ON M5S 3G4 Canada; 2 Department of Chemistry, University of Toronto, 80 St. George Street, Toronto, ON M5S 3G4 Canada; 3 Department of Pharmaceutical Sciences, Leslie Dan Faculty of Pharmacy, University of Toronto, Toronto, ON M5S 3M2 Canada; 4 Department of Materials Science and Engineering, Korea Advanced Institute of Science and Technology (KAIST), 291 Daehak-ro, Yuseong-gu, Daejeon 34141 Republic of Korea

**Keywords:** Solar cells, Quantum dots, Synthesis and processing

## Abstract

Control over carrier type and doping levels in semiconductor materials is key for optoelectronic applications. In colloidal quantum dots (CQDs), these properties can be tuned by surface chemistry modification, but this has so far been accomplished at the expense of reduced surface passivation and compromised colloidal solubility; this has precluded the realization of advanced architectures such as CQD bulk homojunction solids. Here we introduce a cascade surface modification scheme that overcomes these limitations. This strategy provides control over doping and solubility and enables *n*-type and *p*-type CQD inks that are fully miscible in the same solvent with complete surface passivation. This enables the realization of homogeneous CQD bulk homojunction films that exhibit a 1.5 times increase in carrier diffusion length compared with the previous best CQD films. As a result, we demonstrate the highest power conversion efficiency (13.3%) reported among CQD solar cells.

## Abstract (web summary)

It is challenging to realize doping and surface passivation simultaneously in colloidal quantum dot inks. Here Choi *et al*. employ a cascade surface modification approach to solve the problem and obtain record high efficiency of 13.3% for bulk homojunction solar cells based on these inks.

## Introduction

Colloidal quantum dots (CQDs) have attracted intense attention for optoelectronic applications, including light-emitting diodes^1,2^, photodetectors^3,4^, lasers^5^, and photovoltaic devices^6–8^. The broad tunability of their optical and electrical properties through size^9,10^ and surface chemistry modification^6,11^ enables bottom-up design for function and performance. The understanding and manipulation of these properties has triggered continued progress in the performance of CQD devices. In solar cells, improvements in synthesis^12^, surface passivation^13,14^, and device architecture^6,15^ have enabled advances in power conversion efficiency (PCE), which has now reached certified values of 12% in single-junction solar cells^16^.

Major strides in improving the performance of CQD solar cells have been achieved through increasing the carrier diffusion length (*L*_D_), which provides improved charge extraction efficiency^14,16,17^. The diffusion length is determined by the lifetime and the mobility of minority carriers. One strategy for increasing the diffusion length is to minimize the deep electronic trap states at CQD surface. Ligand exchanges enable surface passivation of CQDs, which results in improved carrier transport and longer carrier lifetime in CQD solids^14,18^.

In a complementary strategy, one may architect devices that favor charge transport and extraction in CQD solids by—in a bulk heterojunction—separating photoexcited electrons and holes into distinct phases and then collecting them at charge-selective contacts. This approach results in extended carrier lifetimes, reduced recombination rates, and therefore longer effective diffusion lengths^19,20^. CQD/semiconducting polymer blends^21,22^, or two different CQD materials^20,23^, are material combinations used previously to implement bulk heterojunctions.

A single-bandgap choice in CQD materials can also be used, forming bulk homojunctions in which the two phases are distinguished by their doping^24^. The density of states of ligand/CQD systems is influenced by ligand functionalization (arising from their electron-donating vs. electron-withdrawing character)^6,25–28^, and as a result, the use of different ligands provides another degree of freedom in control over the doping level in CQDs^6,11^.

However, despite their advantages in carrier extraction and transport, CQD bulk homojunction devices are yet to outperform^24^ planar devices due to the difficulty in making both *p*-type and *n*-type CQD inks with complete surface passivation. In particular, previously explored ligand-exchange approaches for *p*-type CQD inks have resulted in surface defects that find their origins in the steric hindrance of the doping ligands^29^, a fact that prevents comprehensive surface coverage. The two ink types also need to be fully miscible with one another. Instability in blend CQD inks leads to aggregation of CQDs and nonuniform morphology in the final films, factors detrimental to optoelectronic device performance.

Here we demonstrate homogeneous CQD bulk homojunction solids through a cascade surface modification (CSM) strategy. The CSM consists of an initial halogenation step of CQD surfaces to attain an initial sufficient passivation, and a subsequent step that reprograms CQD surfaces with functional ligands to control the doping character and solubility properties of the resulting CQD inks. The resulting *p*-type and *n*-type CQDs exhibit a distinct potential difference, which induces a built-in electric field between the constituent classes of CQDs. By controlling the colloidal solubility of the inks, we achieve homogeneous CQD bulk homojunction films, whereas we show that the use of prior ink strategies results in inhomogeneous films as a result of poor miscibility. The homogeneous CQD bulk homojunction films exhibit a 1.5-fold increase in the carrier diffusion length and outperform previously reported CQD solar cells, achieving a record PCE of 13.3%.

## Results

### Materials synthesis and characterization

**Fig. 1:**
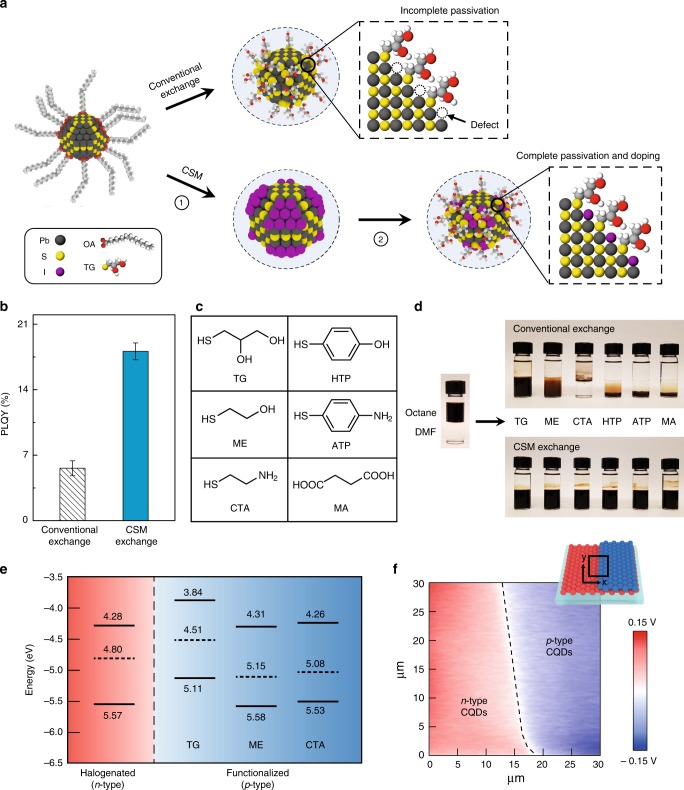
CSM enables tunable doping and passivation of CQD inks. **a** CSM consists of Step 1 (halogenation): the oleic-acid ligands are exchanged with lead halide anions; Step 2 (functionalization): lead halide anions are re-exchanged with the functional ligands to render *p*-type character. For the conventional solution-phase exchange process, the functional ligands are unable to fill the surface due to steric hindrance, which retains surface defects. In the CSM case, the initial halogenation step infiltrate sites not readily accessed by the large organic ligands. **b** PLQY measurements of CQD inks exchanged using conventional vs. CSM methods. TG ligands were used in this particular study. Error bars represent standard deviation calculated from a sample of three CQD inks. **c** Chemical structure of various functional ligands employed herein. **d** Photographs showing phase transfer of CQDs from octane to dimethylformamide (DMF) upon ligand exchange with functional ligands. Conventional exchange exhibits precipitation of CQDs due to low colloidal solubility, while CSM exchange enables to form stable colloids in the DMF phase. **e** The energy levels of the CQD films fabricated using *n*-type CQDs (halogenated) and *p*-type CQDs (functionalized), measured using UPS taken together with UV/visible absorbance. Top edge, dashed line, and bottom edge represent conduction band edge, Fermi level, and valence band edge, respectively. **f** Two-dimensional KPFM potential image of the CQD film. Inset schematic shows the film structure that consists of bottom *n*-type CQD layer (red) and top *p*-type CQD layer (blue). The mapping was performed at the interface between the *n*-type layer and the *p*-type layer (rectangular area). The halogenated CQD inks and CTA-reprogrammed inks were used as *n*-type and *p*-type, respectively.

We synthesized *n*-type and *p*-type CQD inks via the CSM process depicted in Fig. 1a. Initially, CQDs are capped with oleic acid and dispersed in octane. We first proceed with surface halogenation of CQDs with lead halide anions to obtain *n*-type CQD inks, after which the dots are transferred to dimethylformamide (DMF) in which they form a stable colloid. In the second step, we reprogram the CQD surface—rich in lead halide anions—with thiol ligands, introduced to render the CQD inks *p*-type. X-ray photoelectron spectroscopy (XPS) was used to monitor the surface reprogramming of CQD inks. The measurements revealed a bound thiolate peak in XPS S 2p spectra and a strong decrease of the XPS I 3d peak (Supplementary Fig. 1).

To evaluate the degree of surface passivation of CQD inks, we measured the materials’ photoluminescence quantum yield (PLQY). Prior solution-phase ligand-exchange methods give a low PLQY of 6% due to a lack of surface passivation (Fig. 1b). In contrast, the CSM-programmed CQD inks using the same 1-thioglycerol (TG) ligands exhibit a threefold higher PLQY of 18%. This highlights the role of the initial halogenation step to infiltrate sites otherwise inaccessible to bulky organic ligands.

Figure 1c shows the chemical structure of various functional ligands [TG, 2-mercaptoethanol (ME), cysteamine (CTA), 4-hydroxythiophenol (HTP), 4-aminothiophenol (ATP), and malonic acid (MA)]. In contrast with previous ink strategies, which were limited to the use of TG, the CSM method enables use of a wider set of molecules on CQD surfaces, and achieves stable colloids (Fig. 1d), showcasing the versatility of the method.

The surface reprogramming with thiol ligands increases the S/Pb atomic ratio of CQD inks from 0.81 to 1.15 (Supplementary Fig. 1), and we found that this stoichiometric control induces *p*-type characteristics as evidenced by ultraviolet photoelectron spectroscopy (Fig. 1e and Supplementary Fig. 2). The *n*-type CQD inks are tuned to *p*-type after surface reprogramming: the energy difference between the valence band and the Fermi level decreases from 0.77 to 0.6 (TG), 0.43 (ME), and 0.45 (CTA), respectively.

We employed Kelvin probe force microscopy (KPFM) to measure the surface potential difference between *n*-type and *p*-type CQDs to assess whether net doping of each phase was retained following self-assembly to the final CQD solid (Fig. 1f). The inset shows a schematic of the film structure. The two-dimensional KPFM potential image shows an ~0.2-eV change at the interface between *p*-type and *n*-type CQDs, giving evidence of the energy offset in Fermi levels between *n*- and *p*-type CQDs within the thin films. The local variation in surface potential is due to variation in the film thickness over the area studied in KPFM, which is confirmed by atomic force microscopy topographic image (Supplementary Fig. 3).

We also investigated the effect of doping on carrier transport properties using the space charge-limited current (SCLC) method^30^. Hole- and electron-only devices fabricated with *n*-type and *p*-type CQDs, respectively, reveal that the *p*-type CQDs exhibit a higher hole mobility (*μ*_h_ = 1.3 × 10^−3^ V cm^−1^ s^−1^) and lower electron mobility (*μ*_*e*_ = 1.5 × 10^−3^ V cm^−1^ s^−1^) compared with the *n*-type CQDs (*μ*_h _= 8 × 10^−4^ V cm^−1^ s^−1^; *μ*_e _= 3 × 10^−3^ V cm^−1^ s^−1^) (Supplementary Fig. 4), a trend also seen in a prior report^26^.

### Fabrication of homogeneous CQD bulk homojunction films

**Fig. 2:**
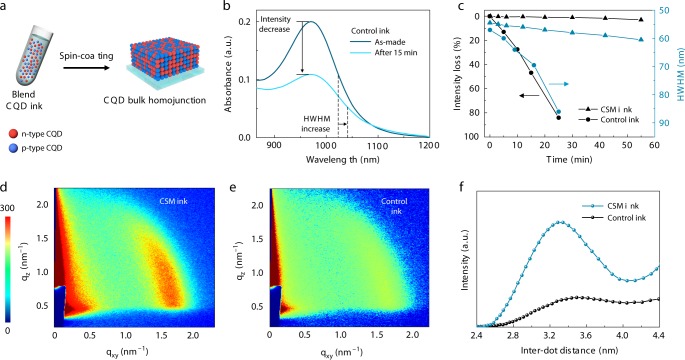
The fabrication of a blend of CQDs to form bulk homojunction films. **a** Blended CQD inks comprising *n*-type and *p*-type CQDs for the fabrication of CQD bulk homojunction films. **b** The absorption spectra of a blend CQD inks (control) in the case of as made and also 15 min later. The absorption spectrum changes rapidly due to the precipitation and heterogeneous fusion of CQDs. **c** The intensity loss and an increase of HWHM in the absorption spectra as a function of time for control vs. CSM-blend CQD inks. **d**–**e** GISAXS two-dimensional pattern of CQD bulk homojunction films made by CSM inks (**d**), and control inks (**e**). Blue color represents lower intensity and red color represents higher intensity. **f** Azimuthally integrated GISAXS intensities reveal more uniform and dense packing of CQDs in the film with CSM inks.

We sought to fabricate CQD bulk homojunction films by using these oppositely doped inks (Fig. 2a). In this step, the solution miscibility of two inks is needed to realize homogeneous CQD films. Precipitation, aggregation, and size polydispersity of CQDs occur when blend inks are not colloidally stable, as a result of heterogeneous fusion between CQDs. This leads to energetic disorder that inhibits carrier transport in the films. CQD fusion and polydispersity are observed as inhomogeneous broadening in the absorption spectra, where the intensity of the bandedge exciton peak will decrease, and its half-width at half-maximum (HWHM) will increase (Fig. 2b). Measuring the absorption spectra over time shows that the mixture of *n*-type and *p*-type CQD inks produced by conventional solution exchange methods (control inks) undergoes rapid degradation of their initial properties (Fig. 2c).

Since it was previously demonstrated that *n*-type CQD inks are dispersed well in butylamine (BTA) due to surface lead halides^16^, we sought to tailor solubility of *p*-type CQD inks by using CSM method to form stable colloids of blend inks in BTA. The colloidal solubility of *p*-type inks is determined by the other functional group in thiol ligands (–L in SH–R–L) because thiols (–SH) bind strongly to the CQD surface. We hypothesized that the strength of the hydrogen bond of the –L termination with respect to BTA would be the key determinant of colloid stability. A competing scenario where –L moieties possessed a stronger hydrogen bond with one another compared with BTA would promote CQD aggregation and colloid precipitation. Only when the strength of the hydrogen bond for L-BTA is balanced or stronger than L–L can *p*-type CQD inks form stable colloids in BTA.

Given the strength of hydrogen bonds between each functional group (COOH–COOH > OH–OH > NH_2_–NH_2_), we reasoned that NH_2_ would be the most well-suited functional group to form stable colloids in BTA because BTA contains NH_2_ group. We then synthesized CSM-programmed CQD inks employing various bifunctional thiol ligands containing different functional groups (COOH, OH, and NH_2_). The experiments revealed that the NH_2_ group (CTA) enables the formation of stable colloids, whereas the OH group (ME) exhibited limited stability, and the COOH groups (3-mercaptopropionic acid, MPA) were insoluble in BTA (Supplementary Fig. 5). We therefore developed stable-blend CQD inks consisting of CTA-reprogrammed *p*-type inks and *n*-type CQD inks (Fig. 2c). Henceforth, we define *p*-type CQD inks as CTA-reprogrammed CQD inks.

To investigate the impact of colloidal stability of inks on the final film formation and morphology, we carried out grazing-incidence small-angle X-ray scattering (GISAXS) measurements. For the CQD bulk homojunction film made from CSM inks, intensity accumulation indicates a hexagonal pattern and in-plane ordering of CQDs (Fig. 2d)^31^. Notably, conventional inks lose packing uniformity and do not show a clear hexagonal pattern in the final CQD solid (Fig. 2e). Azimuthal integration of the diffraction pattern shows an average inter-dot distance of 3.32 nm for CQD bulk homojunction film with CSM inks (Fig. 2f). Comparatively, inter-dot distances of 3.30 nm for the *n*-type CQD film and 3.35 nm for the *p*-type CQD film were found (Supplementary Fig. 6). This agrees with the picture that a substantially homogeneous CQD bulk homojunction film is formed.

### Photophysical properties of CQD bulk homojunction films

**Fig. 3:**
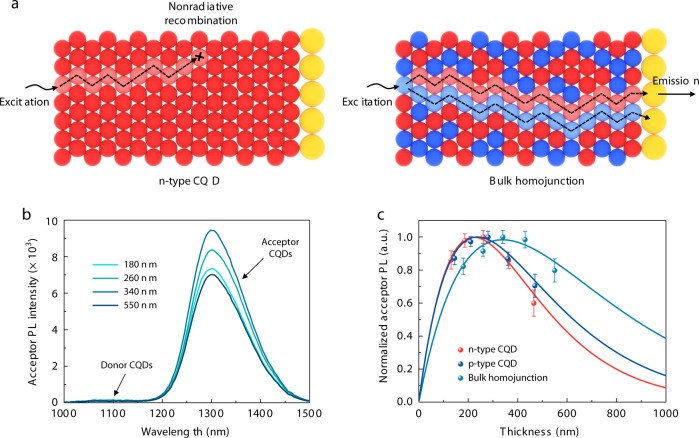
Carrier diffusion length studies for CQD bulk homojunction films. **a** The donor layer is illuminated at one end, generating photoexcited carriers that diffuse to the acceptor layer at the other end. The bulk homojunction provides distinct physical paths for each class of charge carrier, enabling efficient carrier transport with minimized nonradiative recombination. **b** PL intensity with different thicknesses of bulk homojunction (donor) film. **c** Normalized acceptor PL intensity as a function of donor layer thickness (dot). Fits to data (solid line) indicate the enhancement in the carrier diffusion length (dotted line) in bulk homojunction film compared with *n*-type and *p*-type CQD films. The average (symbols) and standard deviation (error bars) are calculated from a sample of three CQD films in each case.

We sought to investigate the carrier diffusion length of CQD bulk homojunction films made from CSM inks. We employed a one-dimensional donor–acceptor scheme^32^ in which incident light excites the top-donor CQD layer (*E*_g_ = 1.3 eV, diffusive layer) and the photoexcited carriers transport to the bottom-acceptor CQD layer (*E*_g_ = 1.0 eV, emitter) where they can recombine radiatively (see the “Methods” section for sample preparation). Given the different solubility properties between the donor CQDs and the acceptor CQDs, we opted to avoid three-dimensional donor–acceptor scheme^33^, because it would be difficult to gauge if the acceptor CQDs would homogeneously mix with the donor CQDs. UPS measurements show that the energy level of the acceptor CQD layer forms a type-I heterojunction with both *n*-type and *p*-type CQDs, making it suitable to be used as quencher in the 1D diffusion length studies (Supplementary Fig. 7). We monitored this recombination as a function of thickness of the donor layer (Fig. 3a). As the thickness of the donor layer is increased, the photoluminescence (PL) intensity of the acceptor layer starts to decrease after a certain thickness is reached (Fig. 3b), when fewer charge carriers reach the acceptor layer due to nonradiative recombination in the diffusive layer. Normalized acceptor PL intensity is plotted as a function of donor layer thickness (Fig. 3c). To evaluate the carrier diffusion length, we fit the data using a 1D diffusion length model^32^. It indicates that the CQD bulk homojunction films show longer carrier diffusion lengths (340 nm) by a factor of 1.5× compared with those of the previous best CQD control films (221 nm), which consist of only *n*-type halogenated CQDs. The optical constants (*n* and *k* values) of the *n*-type and *p*-type CQD solids show similar values to within experimental error (Supplementary Fig. 8). This supports the view that the results obtained from the one-dimensional donor–acceptor diffusion length measurements are not due to different optical parameters among the doped CQD solids.

The orthogonality in the surface chemistry of *p*-type and *n*-type CQD inks in blend solution (their ability to retain their surface ligands when mixed in solution and in solid state), as well as their stability, were also studied to verify the retention of their original band offset. The blend inks show stable PL intensity for an hour, indicating no appreciable evolution in chemistry in the solution phase (Supplementary Fig. 9). We then prepared blend inks consisting of wide *E*_g_ *p*-type inks (*E*_g_ = 870 nm) and narrow *E*_g_ *n*-type inks (*E*_g_ = 1090 nm). Because thiol passivation induces a red shift in the first excitonic peak^34^ (Supplementary Fig. 10), we used it as a proxy to monitor thiol migration from *p*-type to *n*-type species. The addition of CTA in *n*-type inks produces a significant red shift in the first excitonic peak. In contrast, the blend inks do not show a peak shift in the *n*-type CQD population (Supplementary Fig. 11). Taken together, the stable PL intensity of the blend inks, combined with the invariance in the peak position of the *n*-type CQD inks, indicates the retention of chemical orthogonality in, and the colloidal stability of, the blend inks.

**Fig. 4:**
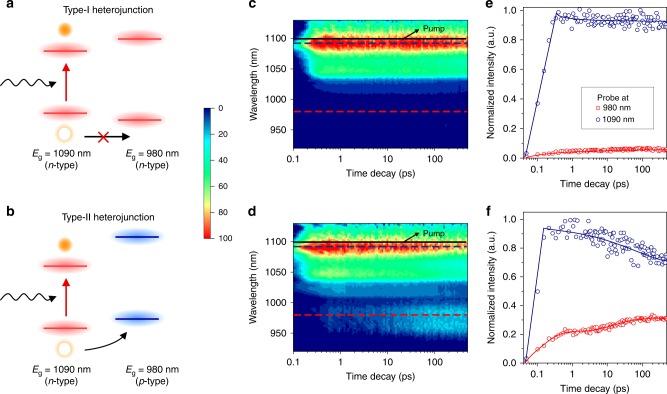
Inter-dot carrier transfer in blend CQD films. **a**, **b** Schematic illustration of photoexcited carrier transfer in *n*-type (*E*_g_ = 980 nm)–*n*-type (*E*_g_ = 1090 nm) blend CQD film (**a**), and *p*-type (*E*_g_ = 980 nm)–*n*-type (*E*_g_ = 1090 nm) blend film (**b**), when narrower *E*_g_ CQDs are selectively populated by pump. **c**, **d** Transient absorption (TA) maps reveal that blending the same type of CQDs does not show carrier transfer due to type-I heterojunction formation (**c**), whereas blending different types of CQDs exhibits hole transfer owing to formation of type-II heterojunction (**d**). **e**, **f** TA spectral kinetic traces of bandedge exciton bleaching signals at each *E*_*g*_ position for (**c**) and (**d**), respectively.

We further investigated the dynamics of charge carrier transfer between the constituent classes of CQDs. The PL intensity of blend films consisting of *n*-type and *p*-type CQDs having the same size and bandgap (*E*_g_ = 950 nm) exhibits strong emission quenching compared with that of purely *n*-type and *p*-type CQD films, a signature of charge carrier transfer in the blend films (Supplementary Fig. 12). We then carried out ultrafast transient absorption (TA) spectroscopy to acquire additional information. First, we prepared blend CQD films consisting of a wide *E*_g_ *p*-type CQD (*E*_g_ = 980 nm) and a narrow *E*_g_ *n*-type CQD (*E*_g_ = 1090 nm) to identify the charge transfer dynamics between the two populations. We selectively populated the narrow-bandgap CQDs using a photoexcitation wavelength of 1100 nm. In this configuration, charge carrier transfer from narrow to wide *E*_g_ CQDs will be indicated by the appearance of an increasing exciton bleach signal in the TA spectra at the corresponding wavelength. Kinetic traces of signal amplitude at wavelengths corresponding to the *p*-type and *n*-type CQD bandedge exciton bleach confirm charge carrier transfer from narrow *E*_g_ to wide *E*_g_ (Fig. 4d, f and Supplementary Fig. 13), evidenced in the rapid increase in the signal at 980 nm and the simultaneous decrease of the signal at 1090 nm. This evidences a functioning type-II heterojunction between *p*-type and *n*-type CQDs (Fig. 4b), indicating that holes are undergoing charge transfer from larger to smaller CQDs^33^. When using instead a mixture of different sized *n*-type CQDs (i.e., the same surface functionalization but different *E*_g_), this produces a type-I heterojunction (Fig. 4a), and there is no appreciable electron or hole transfer between the two spectrally distinct CQDs, evidenced by the lack of signal amplitude exchanged between the two bandedge exciton bleach peaks (Fig. 4c, e).

### Solar cell structure and performance

**Fig. 5:**
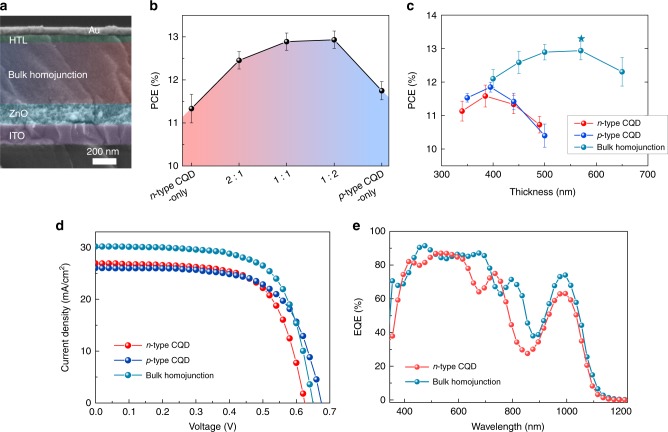
Solar cell performance in devices employing CQD bulk homojunction films. **a** Cross-sectional scanning electron microcopy image of CQD bulk homojunction device. **b** PCE of bulk homojunction devices with different mass ratios of *p*-type and *n*-type CQDs. The thickness of the active layer for the *n*-type CQD devices, *p*-type CQD devices, and bulk homojunction devices are ~390, 400, and 580 nm, respectively. **c** Thickness-dependent PCE for bulk homojunction devices and *n*-type CQD devices (control). Bulk homojunction devices enable the use of much thicker optimized CQD films compared with the case of *n*-type CQD devices. **d** Current–voltage characteristics under AM1.5 illumination for *n*-type CQD device, *p*-type CQD device, and CQD bulk homojunction device at optimum film thickness. **e** External quantum efficiency of *n*-type CQD device and bulk homojunction device at optimum film thickness. The average (symbols) and standard deviation (error bars) are calculated from a sample of six to twelve devices.

Given these promising properties of CQD bulk homojunction films, we pursued the realization of CQD solar cells with enhanced performance. We employed CQD bulk homojunction films as the active layer in a conventional CQD solar cell structure: ITO/ZnO as the electron-accepting layer/CQD film as the active layer/thin CQD film treated with 1,2-ethanethiol (EDT) as hole-transport layer/Au (Fig. 5a). We note that *p*-type CQD inks cannot be employed as the HTL in CQD devices due to their similar solubility properties to those of the *n*-type CQD inks. We first sought to optimize the *p*-type to *n*-type CQD ratio and obtained our best PCE using a 2:1 mass ratio (Fig. 5b). We then explored the PCE as a function of thickness. Notably, the bulk homojunction devices exhibit a substantially greater optimized thickness (580 nm) compared with the devices based on *n*-type CQDs (390 nm) and *p*-type CQDs (400 nm) (Fig. 5c), which is in good agreement with our observations of longer carrier diffusion lengths in the bulk homojunction films. This was accompanied by an enhanced short-circuit current density (*J*_sc_) without compromise to open-circuit voltage (*V*_oc_) and fill factor (FF), and as a result, it led to superior PCE. Using this architecture, we achieved an AM1.5 PCE of 13.3% through the combination of *V*_oc_ of 0.65 V, *J*_sc_ of 30.2 mA/cm^2^, and FF of 68% (Fig. 5d). The AM1.5 PCE from an accredited laboratory (Newport) shows a value of 12.47 ± 0.33%, the highest certified PCE reported for CQD solar cells (Supplementary Fig. 14). The devices with *p*-type CQDs exhibit higher *V*_oc_ compared with the devices with *n*-type CQDs. The downshifted Fermi level of *p*-type CQDs increases the built-in potential of devices, accounting for the higher *V*_oc_^35–37^. Statistical data of the bulk homojunction devices show the reproducibility of these efficiencies (Supplementary Fig. 15). External quantum efficiency (EQE) measurements confirm the high *J*_sc_ of the bulk homojunction device (30 mA/cm^2^) compared with the control device (26.8 mA/cm^2^) (Fig. 5e). We further tested the stability of the CQD bulk homojunction devices. They retained 87% of their initial PCE following 110 h of device operation at their maximum power point under AM1.5 G illumination in an N_2_ atmosphere (Supplementary Fig. 16). We made larger-area (1.1 cm^2^) devices using CQD bulk homojunctions (Supplementary Fig. 17) and obtained similar *V*_oc_ and *J*_sc_ values. A somewhat lower PCE in large-area devices comes from a lower FF related to the series resistance of the transparent conductive oxide^16^.

## Discussion

This work introduces a way to realize the homogeneous bulk homojunction structure in CQD solids. It is achieved via development of a CSM ligand-exchange strategy that enables the synthesis of *p*-type and *n*-type CQD inks that combine excellent surface passivation with miscibility for stable mixed-dot colloids. The CQD bulk homojunction films show an increase in carrier diffusion length compared with the state-of-the-art planar CQD films owing to improved separation and transport of photoexcited carriers through distinct physical paths. This is supported by observations of ultrafast charge transfer between *n*- and *p*-type domains as probed using transient absorption spectroscopy. The resulting solar cells exhibit the highest power conversion efficiency reported in CQD photovoltaic devices (13.3%).

## Methods

### CQD synthesis

Oleic-acid-capped PbS CQDs at 950 nm (1.31 eV) were synthesized based on a previous report^38^. Lead(II) oxide (0.9 g), oleic acid (3 mL), and octadecene (20 mL) were mixed in a three-neck flask and heated to 120 °C under vacuum for 2 h and then filled with N_2_. A stock solution, 0.24 mL of hexamethyldisilathiane dissolved in 8 mL of octadecene, was then injected rapidly into the flask for PbS CQD synthesis. The CQD solution was then slowly cooled to room temperature. Acetone was added to precipitate the QD solution, and the precipitate was then redispersed in toluene. The CQDs were further purified twice by adding a mixture of acetone and methanol. Finally the QDs were dissolved in octane (50 mg mL^−1^).

### CSM process and film fabrication

For both *n*-type and *p*-type CQD inks, PbS CQDs at 950 nm were used. The first exchange was carried out using a modified previous method^14^. Precursor solution was prepared by dissolving lead halides (lead iodide 0.1 M and lead bromide 0.02 M) and NH_4_Ac (0.055 M) in dimethylformamide (DMF). A 5 mL of CQD solution dissolved in octane (7 mg mL^−1^) was added to 5 mL of precursor solution. Then the solution was mixed vigorously for 1–2 min until CQDs were transferred to DMF phase. The octane phase was discarded and DMF solution was washed with octane three times. The DMF solution was precipitated by adding toluene and dried in vacuum. The CQD solids were redispersed in butylamine (BTA). These CQD inks were used as *n*-type CQDs. To produce *p*-type inks, the above DMF solution was further treated with the second surface modification. Thiol solution was prepared by dissolving cysteamine in DMF (0.1 M). The thiol solution was slowly dropped into the DMF solution with gentle stirring (range of 100–400 μL) and precipitated by adding toluene. The CQD solids were dried in vacuum and redispersed in butylamine (BTA). The CQD inks were deposited on a substrate by single-step spin coating to achieve CQD films. The process was carried out under ambient air conditions.

### CQD solar cell fabrication

The ZnO nanoparticles were synthesized using a published method^6^. The ZnO nanoparticles were spin-cast on an ITO substrate at 3000 r.p.m for 30 s. Then CQD inks were spin-cast onto the ZnO/ITO substrate. The blend CQD inks (mixture of *n*-type and *p*-type CQD inks) were used to fabricate bulk homojunction devices. The CQD films were annealed at 70 °C for 5 min in N_2_-filled glove box. Two PbS–EDT layers were then deposited as a hole-transport layer. Oleic-acid-capped CQDs were spin-cast, and then soaked with 0.01 vol% 1,2-ethanedithiol in propionitrile solution for 30 s, followed by three repetitions of washing using propionitrile. Finally, 120 nm of Au was deposited via e-beam evaporation as the top electrode. We note that the use of propionitrile provides higher device performance compared with acetonitrile that was used in prior works^14,16^.

### Solar cell measurements

The active area (0.049 cm^2^) was determined by placing an aperture between the devices and the AM1.5 solar simulator (Sciencetech class A). Current–voltage (*I*–*V*) characteristics were measured with the aid of a Keithley 2400 source measuring unit under simulated AM1.5 illumination. Devices were tested under a continuous nitrogen flow. The *I*–*V* curves were scanned from −0.70 to +0.1 V at 0.02-V interval steps without wait time between voltage steps. The spectral mismatch was calibrated using a reference solar cell (Newport). EQE spectra were taken by subjecting the solar cells to chopped (220 Hz) monochromatic illumination (400-W Xe lamp passing through a monochromator and appropriate cutoff filters). Newport 818-UV and Newport 838-IR photodetectors were used to calibrate the output power. The response of the cell was measured with a Lakeshore preamplifier feeding into a Stanford Research 830 lock-in amplifier at short-circuit conditions.

### Transient absorption measurements

Femtosecond pulses at 1030 nm with a 5-kHz repetition rate were produced using a regeneratively amplified Yb:KGW laser (PHAROS, Light Conversion). A portion of the beam was used to pump an optical parametric amplifier (ORPHEUS, Light Conversion) to serve as a narrowband pump tuned between 1000 and 1200 nm. The other portion of the beam power was focused into a sapphire crystal to generate a white-light supercontinuum probe (900–1300-nm window with various optical filters). Both pulses were directed into a commercial transient absorption spectrometer (Helios, Ultrafast). A time window up to 8 ns was obtained by delaying the probe pulse relative to the pump pulse. The time resolution of these experiments was ~300 fs (pulse duration of the pump pulse). All experiments were performed with fluence less than or equal to ~600 μJ cm^−2^ to minimize Auger recombination^33^.

### Grazing-incidence small-angle X-ray spectroscopy (GISAXS)

Grazing-incidence small-angle X-ray spectroscopy (GISAXS) measurements were conducted at the Hard X-ray MicroAnalysis (HXMA) beamline of the Canadian Light Source (CLS). An energy of 17.998 keV (*λ* = 0.6888 Å) was selected using a Si (111) monochromator. Patterns were collected on a SX165 CCD camera (Rayonix) placed at a distance of 157 mm from the sample. A lead beamstop was used to block the direct beam. Images were calibrated using LaB6 and processed via the Nika software package^39^ and the GIXSGUI MATLAB plug-in^40^.

### One-dimensional carrier diffusion length measurements

For the acceptor CQD layer (*E*_g_ = 1.0 eV), 5 mL of oleic-acid-capped CQD solution dissolved in octane (7 mg mL^−1^) was added to 5 mL of precursor solution (lead iodide 0.1 M and lead bromide 0.02 M, and NaAc 0.055 M in DMF)^41^. Then the solution was mixed vigorously for 5 min until CQDs were transferred to the DMF phase. The octane phase was discarded and the DMF solution was washed with octane three times. The DMF solution was precipitated by adding toluene. The supernatant was removed, and the precipitated material was dried in vacuum. The CQD solids were redispersed in a mixture of BTA:DMF (4:1). The acceptor CQD inks were spin-coated on glass substrates with a thickness of 50–60 nm. Then, the donor CQD layer (*E*_g_ = 1.3 eV) was deposited on top of the acceptor CQD layer using *n*-type CQD inks, *p*-type CQD inks, or blend CQD inks (*n*-type:*p*-type = 1:1). We note that the deposition of the donor CQD inks does not redisperse the underlying CQD acceptor layer: the CQD acceptor layer does not redisperse in butylamine solvent (Supplementary Fig. 18).

Samples were illuminated through the donor CQD layer side using a monochromated Xe lamp at 400-nm wavelength. The normalized PL intensity as a function of donor CQD layer thickness is fit using the equation:^32^

1 $${\mathrm{PL}}_{{\mathrm{acceptor}}} = - \frac{\alpha }{{\alpha ^2L_{\mathrm{d}}^2/\tau - 1/\tau }}\left( {\frac{1}{{L_{\mathrm{d}}}}e^{ - d/L_{\mathrm{d}}}\frac{{e^{d/L_{\mathrm{d}}} - e^{ - \alpha d}}}{{e^{ - d/L_{\mathrm{d}}} - e^{d/L_{\mathrm{d}}}}} + \alpha e^{ - \alpha d} - \frac{1}{{L_{\mathrm{d}}}}e^{d/L_{\mathrm{d}}}\frac{{e^{ - d/L_{\mathrm{d}}} - e^{ - \alpha d}}}{{e^{d/L_{\mathrm{d}}} - e^{ - d/L_{\mathrm{d}}}}}} \right)$$

where *L*_d_ is the carrier diffusion length, *d* is the thickness of donor CQD layer, *α* is the absorption coefficient, and *τ* is the carrier lifetime.

### Other characterization

Photoluminescence measurements were carried out using a Horiba Fluorolog Time Correlated Single Photon Counting system equipped with UV/Vis/NIR photomultiplier tube detectors, dual-grating spectrometers, and a monochromatized xenon lamp excitation source. Optical absorption measurements were carried out in a Lambda 950 UV–Vis–IR spectrophotometer. XPS measurements were carried out using a Thermo Scientific K-Alpha system, with a 75-eV pass energy, and binding energy steps of 0.05 eV. All signals are normalized to Pb. Atomic force microscopy and scanning Kelvin probe microscopy were done using an Asylum Research Cypher AFM. Samples were electrically grounded and AC160-R2 silicon probes with a titanium–iridium coating were used. Imaging was done in tapping mode and a nap pass was done to measure the contact potential difference. Spectroscopic ellipsometry was performed using a Horiba UVISEL Plus Extended Range ellipsometer with an ~200-ms integration time, a 10-nm step size, and an ~1-mm-diameter spot size. Three incident angles (60, 65, and 70°) were used. Soda-lime glass slides were used as substrates for each individual material, with their back sides covered with opaque adhesive tape to eliminate back reflections. Fitting was performed using the Horiba DeltaPsi2 software. Dispersion functions composed of four Voigt oscillators achieved fits with χ^2^ < 1.

### Reporting summary

Further information on research design is available in the Nature Research Reporting Summary linked to this article.

## Supplementary information

Supplementary Information

Solar Cells Reporting Summary

## Data Availability

The data that support the findings of this study are available from the corresponding authors upon reasonable request.
