# Supplementary material for: Cascade surface modification of colloidal quantum dot inks enables efficient bulk homojunction photovoltaics
Source: Nat Commun. 2020 Jan 3;11:103. doi: 10.1038/s41467-019-13437-2 (PMC6941986; doi:10.1038/s41467-019-13437-2)
Supplement: Supplementary file 1 — Supplementary Information [file 41467_2019_13437_MOESM1_ESM.pdf]

Cascade Surface Modification of Colloidal Quantum Dot Inks Enables Efficient Bulk Homojunction Photovoltaics

Min-Jae Choi[1], F. Pelayo García de Arquer[1], Andrew H. Proppe[1,2], Ali Seifitokaldani[1], Jongmin Choi[1], Junghwan Kim[1], Se-Woong Baek[1], Mengxia Liu[1], Bin Sun[1], Margherita Biondi[1], Benjamin Scheffel[1], Grant Walters[1], Dae-Hyun Nam[1], Jea Woong Jo[1], Oleksandr Voznyy[1], Olivier Ouellette[1], Sjoerd Hoogland[1], Shana O. Kelley[2,3], Yeon Sik Jung[4]*, and Edward. H. Sargent[1]*

[1]*Department of Electrical and Computer Engineering, University of Toronto, 10 King's College Road, Toronto, Ontario M5S 3G4, Canada*

[2]*Department of Chemistry, University of Toronto, 80 St. George Street, Toronto, Ontario M5S 3G4, Canada*

[3]*Department of Pharmaceutical Sciences, Leslie Dan Faculty of Pharmacy, University of Toronto, Toronto, Ontario, Canada, M5S 3M2*

[4]*Department of Materials Science and Engineering, Korea Advanced Institute of Science and Technology (KAIST), 291 Daehak-ro, Yuseong-gu, Daejeon, Republic of Korea*

*E-mail: ted.sargent@utoronto.ca (E.H.S.), ysjung@kaist.ac.kr (Y.S.J.)

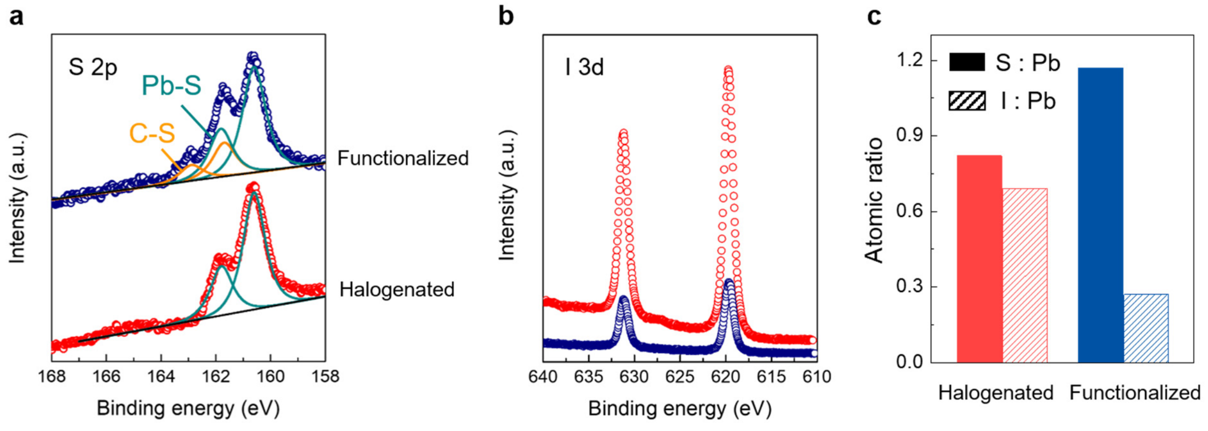

Supplementary Figure 1. a-b, X-ray photoelectron spectroscopy (XPS) S 2p spectra (**a**), and I 3d spectra (**b**) of CQD inks after surface halogenation (red) and surface functionalization with TG ligands (blue). After functionalization, bound thiolate (C-S) peak appears as marked in orange line in (**a**), while iodine 3d peaks decrease strongly (**b**). **c**, S to Pb and I to Pb atomic ratio of CQDs.

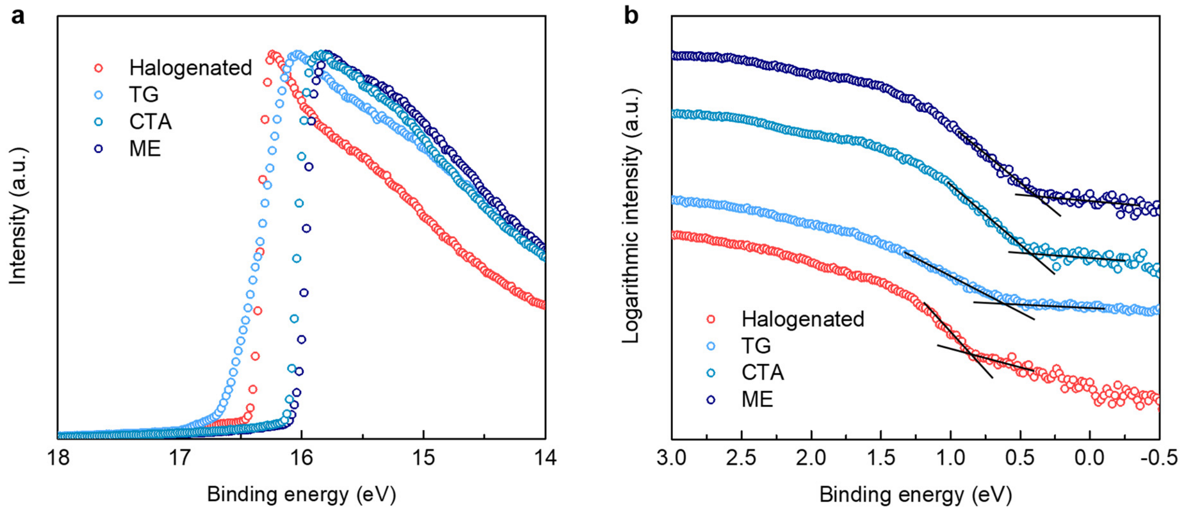

Supplementary Figure 2. a-b, UPS spectra in the secondary cutoff region (**a**), and semilogarithmic valence band region (**b**) for CQD inks with different surface: halogenated and reprogrammed with TG, CTA, and ME ligands.

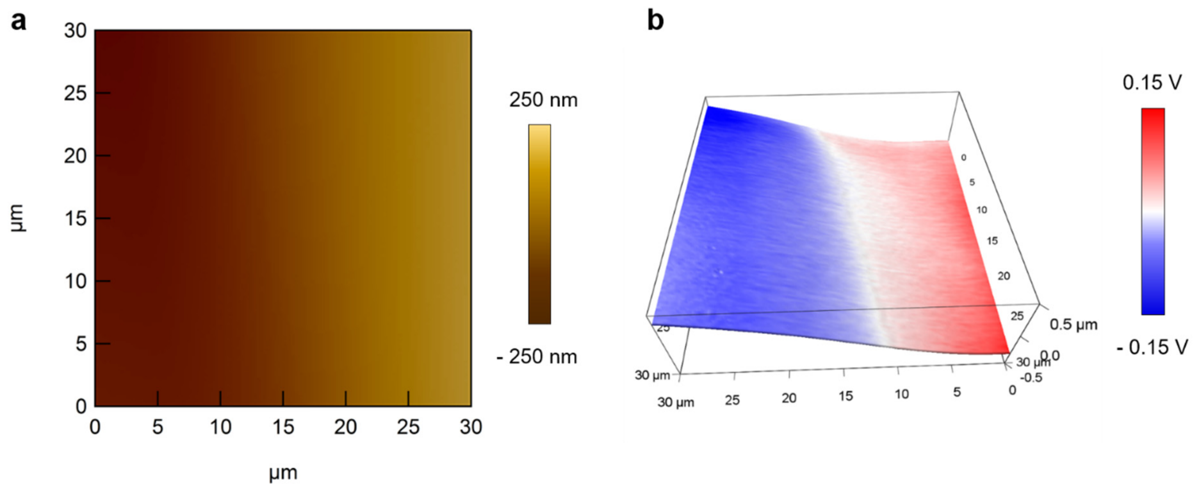

Supplementary Figure 3. a-b, Atomic force microscopy topographic image (**a**), and 3-dimensional KPFM potential image (**b**) at the boundary between *p*-type CQDs (top layer) and *n*-type CQDs (bottom layer). The sharp decrease of surface potential is observed at the interface due to the Fermi level difference.

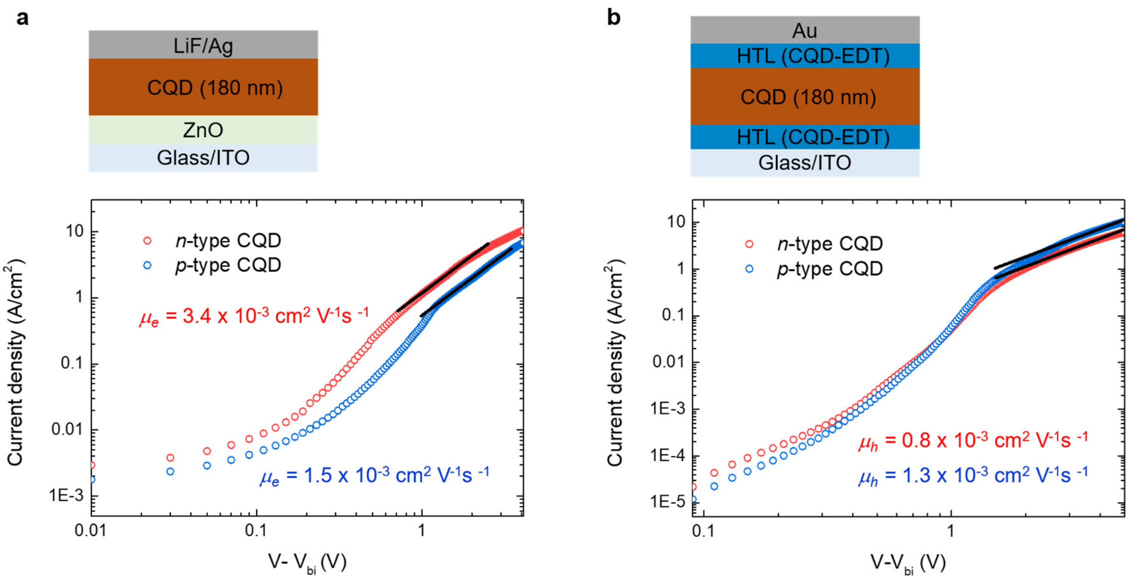

Supplementary Figure 4. a, The structure of electron-only SCLC diodes and electron mobility of *n*-type CQDs (red) and *p*-type CQDs (blue). **b**, The structure of hole-only SCLC diodes and hole mobility of *n*-type CQDs (red) and *p*-type CQDs (blue). Black line indicates SCLC regime following Mott–Gurney's law ($J \sim V^2$). The below equation is used to calculate the mobility:

$$J_{SCLC} = \frac{9}{8}\varepsilon_0 \varepsilon_r \mu_{SCLC} \frac{V^2}{L^3}$$

where ε_0 and ε_r are the vacuum and relative dielectric permittivities, V is the applied voltage, and L is the film thickness. A value of 18.7 is used for dielectric constant of PbS CQD films[1].

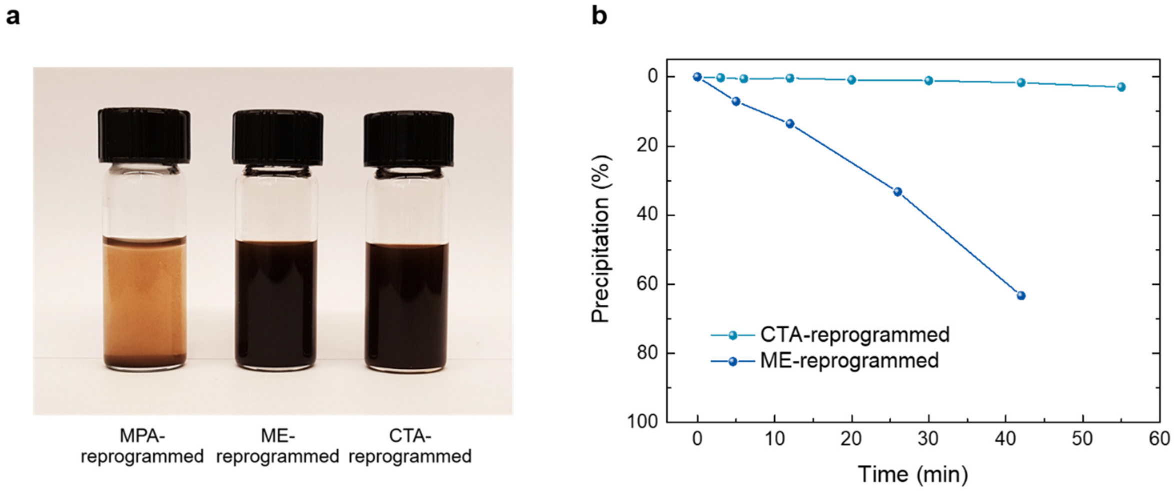

Supplementary Figure 5. a, Photographs of CSM-programmed CQD inks dissolved in BTA with different functional ligands. **b**, The degree of precipitation of CQD inks as a function of time when they are dissolved in BTA.

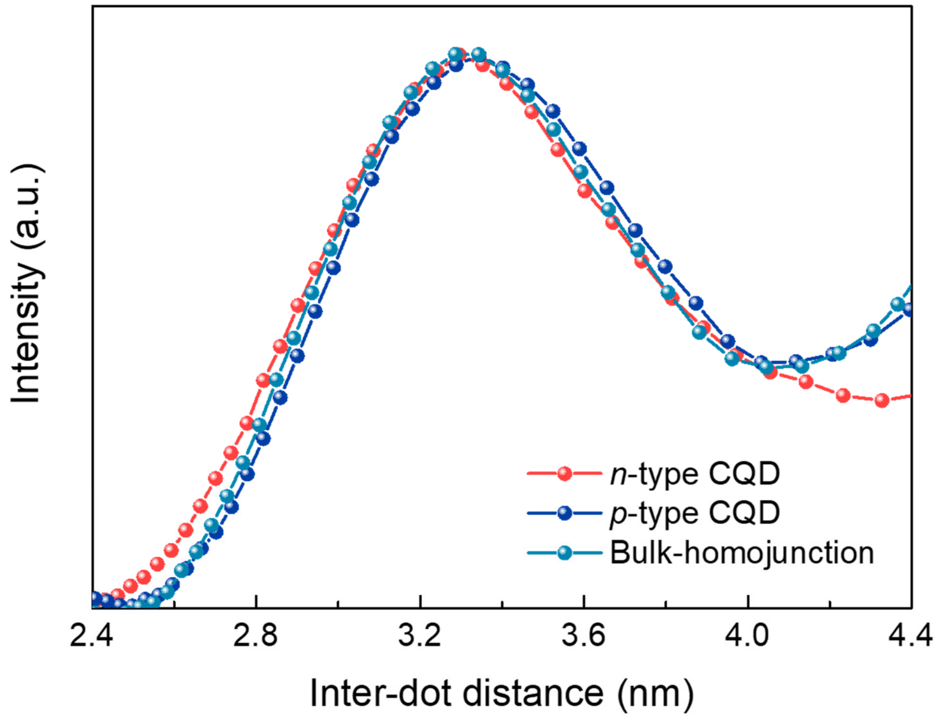

Supplementary Figure 6. Azimuthal integration of the diffraction pattern shows an average inter-dot distance of CQD films fabricated by pure *n*-type CQDs (3.30 nm), pure *p*-type CQDs (3.35 nm), and CQD bulk homojunction (3.32 nm).

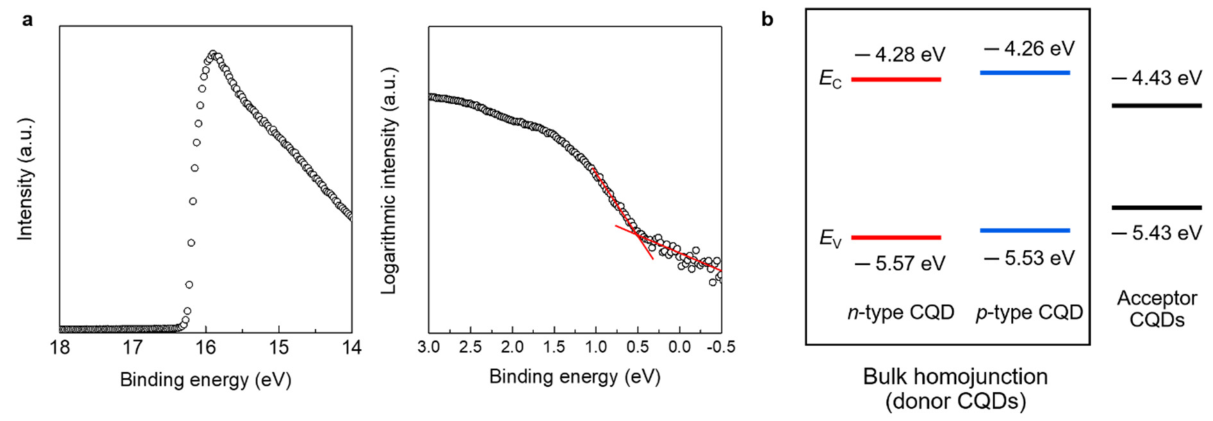

Supplementary Figure 7. a, UPS spectra in the secondary cutoff region and semilogarithmic valence band region for acceptor CQD film (E_g = 1.0 eV) for carrier diffusion length measurement. **b**, Energy level diagram of donor CQD film (E_g = 1.3 eV, *n*-type CQD and *p*-type CQD) and acceptor CQD film.

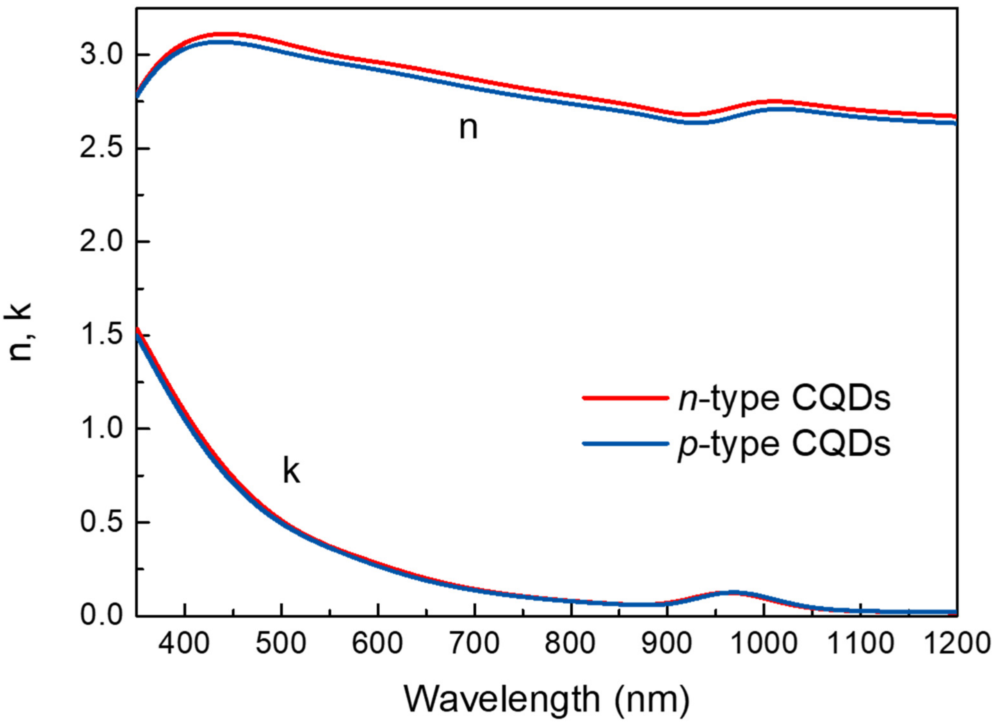

Supplementary Figure 8. The *n* and *k* values for *n*-type CQD film and *p*-type CQD film (CTA-reprogrammed).

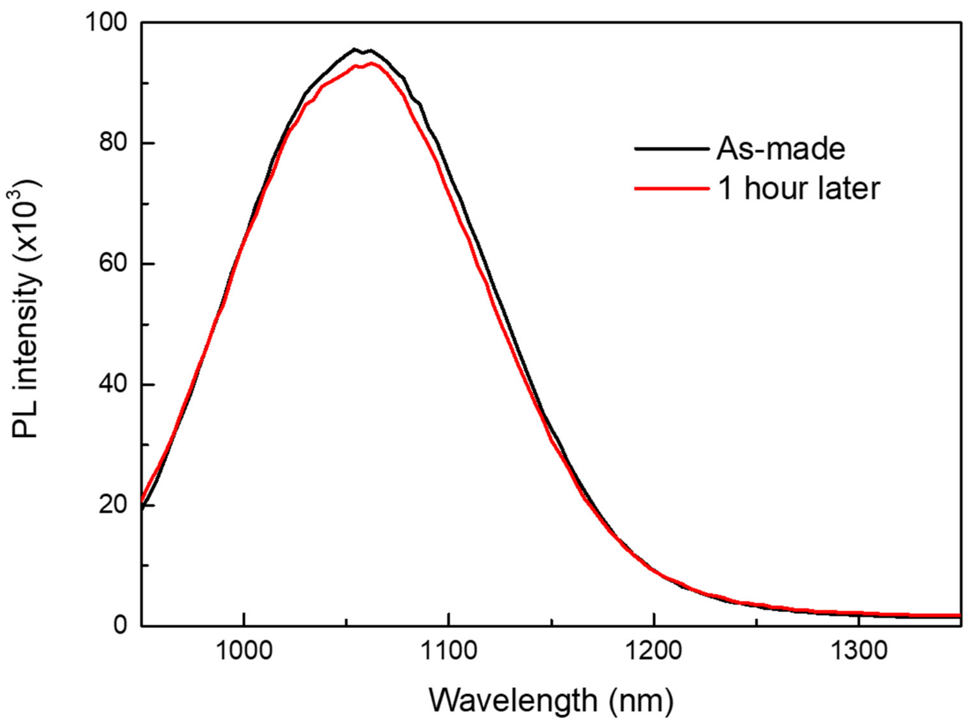

Supplementary Figure 9. Photoluminescence intensity of blend CSM inks consisting of *n*-type (halogenated) and *p*-type CQDs (CTA-reprogrammed). Consistent PL intensity of blend CQD inks indicates chemical orthogonality of blend CQD inks.

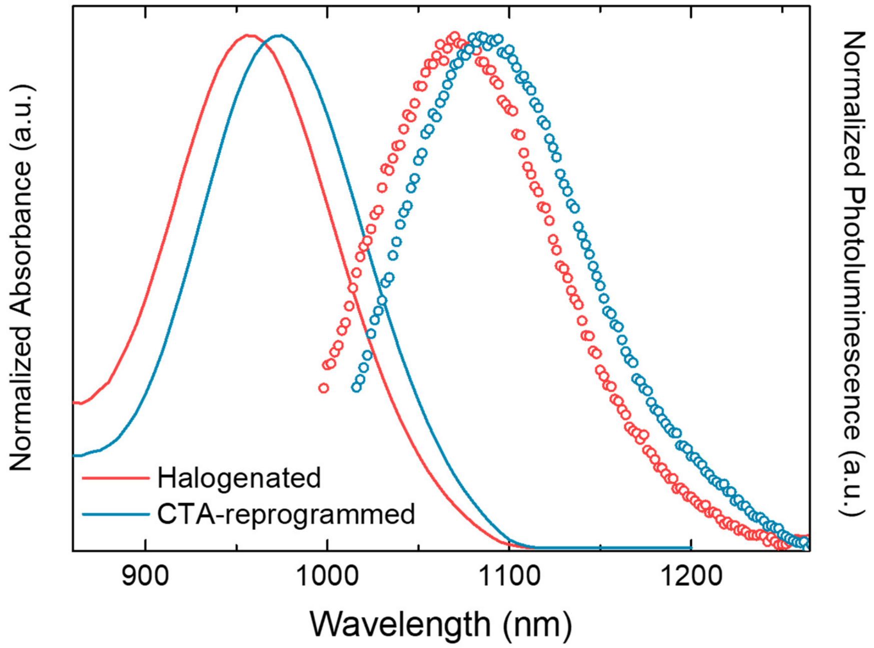

Supplementary Figure 10. Absorbance and photoluminescence of CQD films prepared by CQD inks after surface halogenation and CTA-reprogramming, respectively. Because of S attachment, the optical band gap of CQDs slightly decreases after CTA-reprogramming.

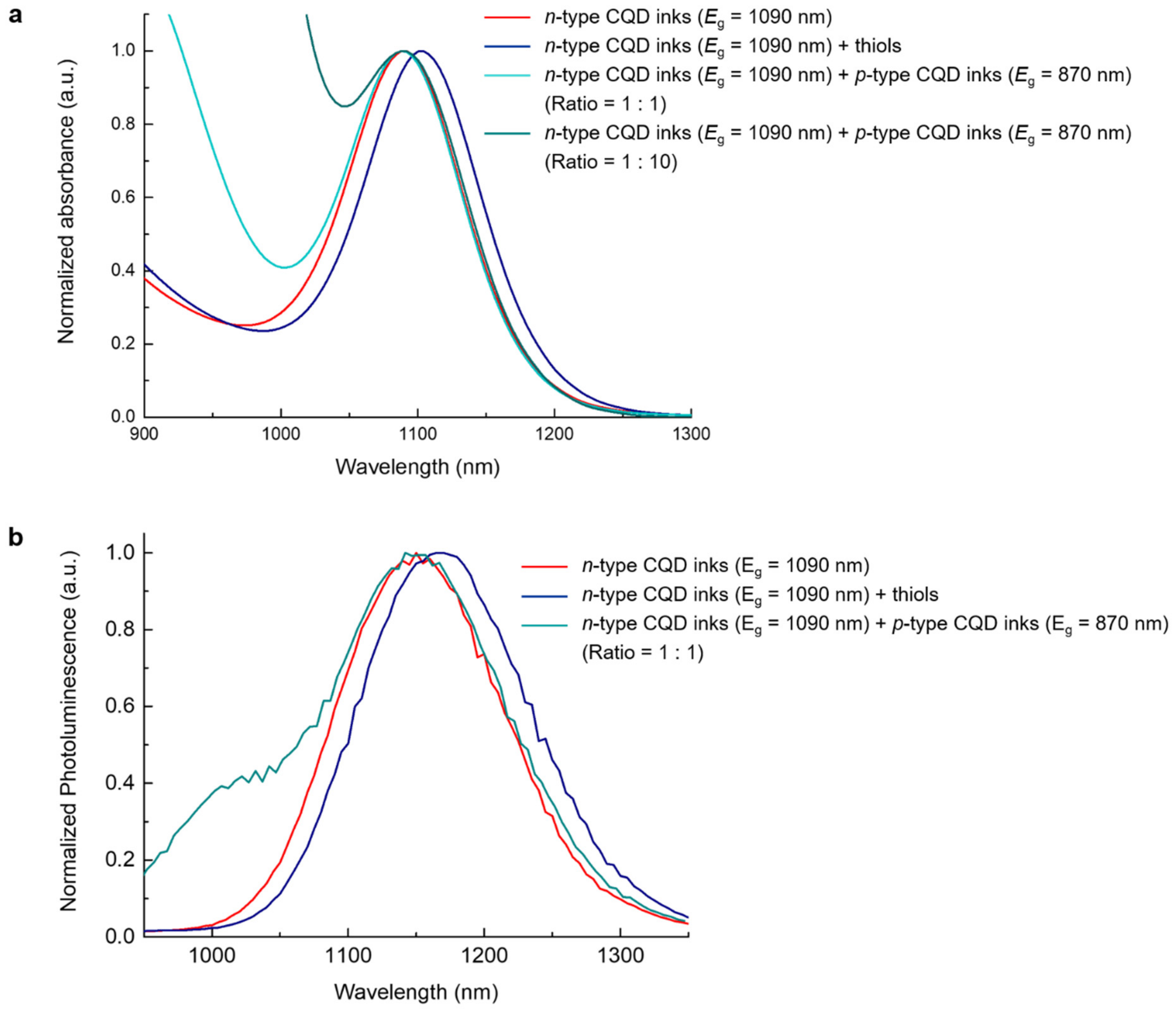

Supplementary Figure 11. a-b, Chemical orthogonality studies of blend CQD inks using absorbance (**a**) and PL (**b**) measurement. Both in absorbance and PL, there are no shift of excitonic peak of *n*-type CQDs in blend CQD inks while direct injection of thiols renders red-shift. It clearly indicates thiol ligands at *p*-type CQDs do not move to *n*-type CQDs, proving chemical orthogonality in blend CQD inks.

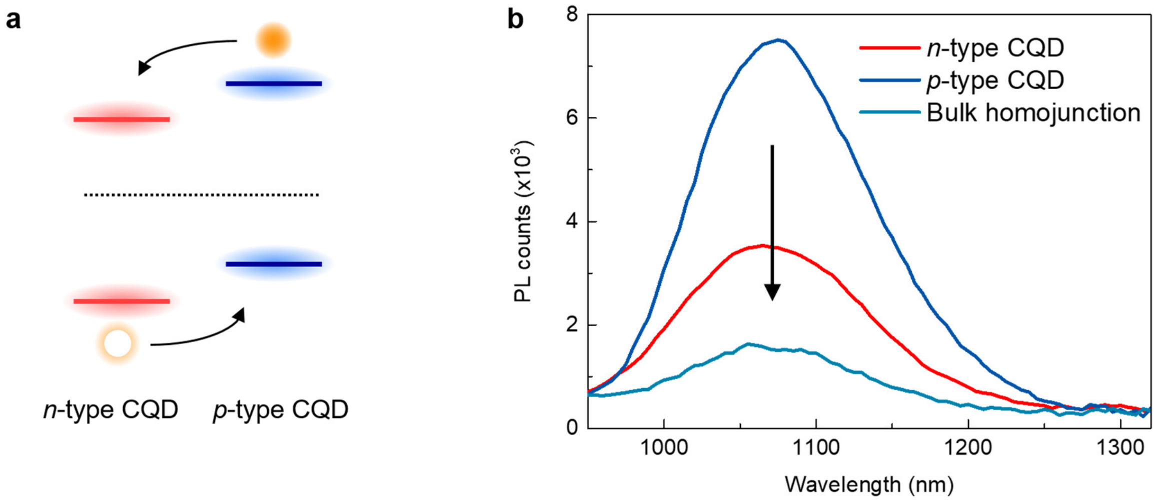

Supplementary Figure 12. **a,** Schematics of carrier transfer between *n*-type CQD and *p*-type CQD in the CQD bulk homojunction film. **b,** The PL intensity of *n*-type CQD, *p*-type CQD, and bulk homojunction film (1:1 ratio).

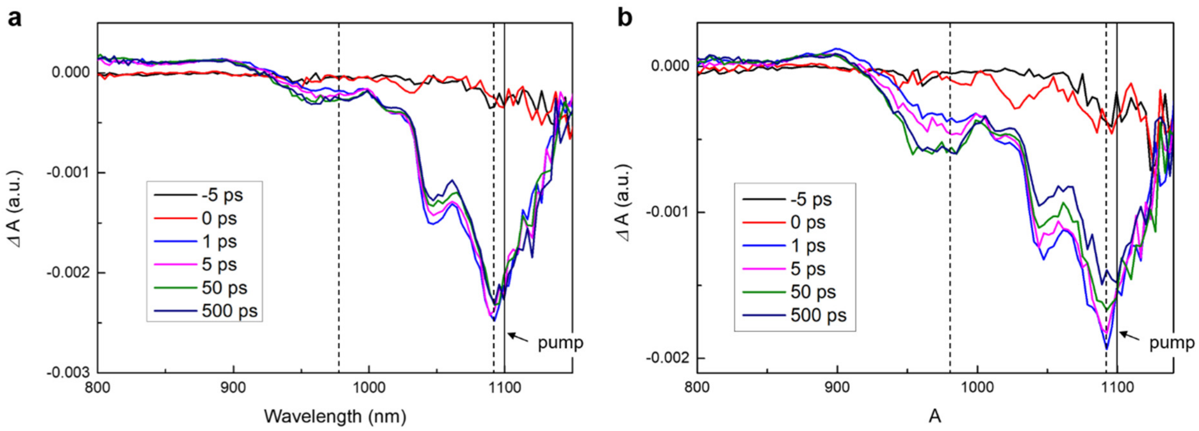

Supplementary Figure 13. Transient absorption spectra of blend CQD films consisting of *n*-type (E_g = 870 nm) / *n*-type (E_g = 1090 nm) (**a**), and *p*-type (E_g = 870 nm) / *n*-type (E_g = 1090 nm) (**b**). The narrow bandgap CQDs (E_g = 1090 nm) are selectively populated using a photoexcitation wavelength of 1100 nm. Rapid increase of absorption at 980 nm in (**b**) indicates hole transfer from *n*-type CQDs to *p*-type CQDs.

Newport	ACCREDITED<br>Calibration Cert. # 2893.01	**Technology and Application Center**<br>**PV Lab**<br>Newport Calibration Cert. # 1977

DUT S/N: 7187113 MJC-1 H1
Newport Calibration #: 1977
Manufacturer: University of Toronto, Dept. of ECE, Ted Sargent Group
Material (single junction): Colloidal quantum dots
Measurement Date: 14-Nov-2018
Temperature Sensor: TC-K
Environmental conditions at the time of calibration: Temperature: 24 ± 3 °C; Humidity: 40 ± 10 %

The above DUT has been tested using the following methods to meet the ISO 17025 Standard by the PV Lab at Newport Corporation. Quoted uncertainties are expanded using a coverage factor of $k = 2$ and expressed with an approximately 95% level of confidence. Measurement of total irradiance is traceable to the World Radiometric Reference (WRR) and all other measurements and uncertainties are traceable to NIST and the International System of Units (SI). The performance parameters reported in this certificate apply only at the time of the test, and do not imply future performance.

* **Designated area defined by opaque thin metal aperture mask.**

† **Reported performance parameters are average of reverse (open-circuit → short-circuit) and forward (short-circuit → open-circuit) IV sweep measurement at a sweep rate of 670 mV/s, and *do not represent the device behavior under quasi-steady-state conditions*. At this sweep rate, hysteresis of +/- 0.36% of PCE was observed.**

Efficency [%]	12.47† ± 0.33	V_oc [V]	0.6523 ± 0.0045	I_sc [A]	0.001363 ± 0.000029
P_max [mW]	0.593 ± 0.015	V_max [V]	0.5110 ± 0.0057	I_max [A]	0.001161 ± 0.000026
FF [%]	66.8 ± 0.8	Area [cm^2]	0.0476* ± 0.0002	M	0.997 ± 0.015

Methods:
I-V: ASTM E948-16 *Standard Test Method for Electrical Performance of Photovoltaic Cells Using Reference Cells Under Simulated Sunlight*
QE: ASTM E1021-15 *Standard Test Method for Spectral Responsivity Measurements of Photovoltaic Devices*

Standard Reporting Conditions:
Spectrum: AM1.5-G (ASTM G173-03/IEC 60904-3 ed. 2)
1000.0 W/m ^{2} at 25.0°C

Secondary Reference Cell:
Device S/N: 10510-0054 KG1
Device Material: mono-Si
Window Material: KG1
Certification: National Renewable Energy Laboratory
A2LA accreditation certificate # 2236.01
ISO Tracking #: 1937
Certified short circuit current (I_{sc}) under standard reporting conditions (SRC): 57.21 mA
Calibration due date: 29-Sep-19

Solar Simulator:
Spectrum: Newport Corporation filename *Sol3A_Spectroradiometer_Scan_0202.xls*
Total irradiance: 1000 W/m^2 based on I_{sc} of the above Secondary Reference Cell

Quantum Efficiency for DUT:
Newport Corporation filename *QE 7187113 MJC-1 H1-5, 0,57 mA WLB.xls*
Spectral mismatch correction factor: M = 0.997 ± 0.015

DUT Calibration Procedures:
Newport Corporation document WI1 (EQE).docx
Newport Corporation document Area Measurement WI2 (Area).docx
Newport Corporation document WI3 (IV.Sweep).docx

Cal Cert V1.8	Issue Date: Nov 19, 2018	Page 2 of 2
	Reviewed and Approved by: Geoffrey Wicks (Geoffrey.Wicks@Newport.com)	
This certificate to be reproduced in part only with written permission from the Newport PV Laboratory		

Supplementary Figure 14. Certified record AM1.5 PCE to date for CQD solar cells. This device is made by CQD bulk homojunction films made from CSM inks.

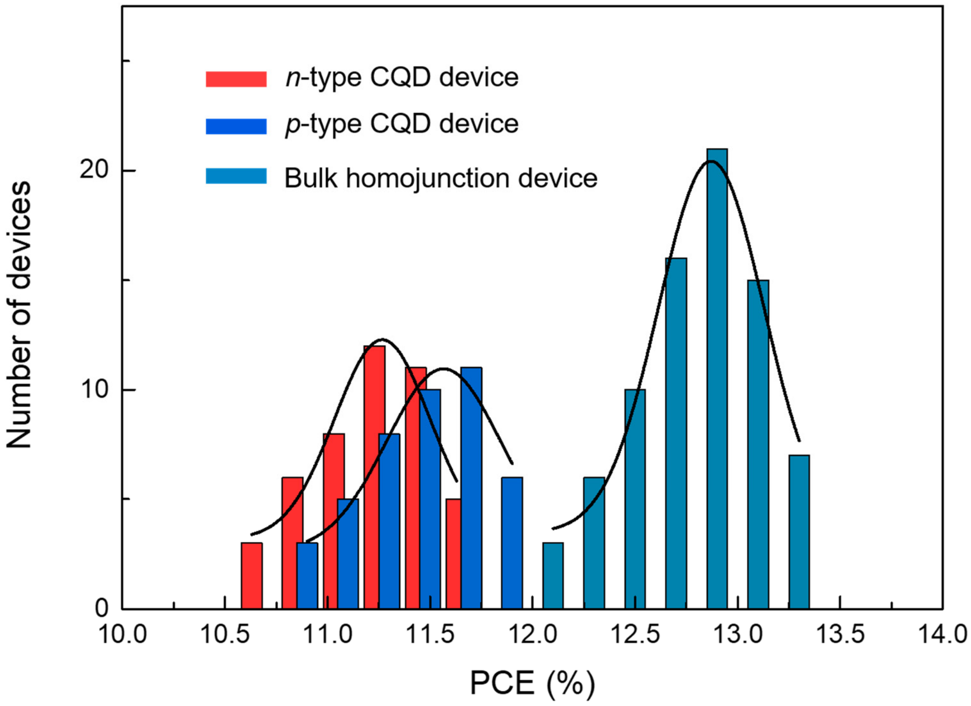

Supplementary Figure 15. Histograms of PCE for *n*-type CQD devices, *p*-type CQD devices, and CQD bulk homojunction devices with Gaussian fits overlaid. The bulk homojunction devices exhibit reproducible high efficiencies. 45 devices (*n*-type), 43 devices (*p*-type), and 78 devices (bulk homojunction) are used for histograms, respectively.

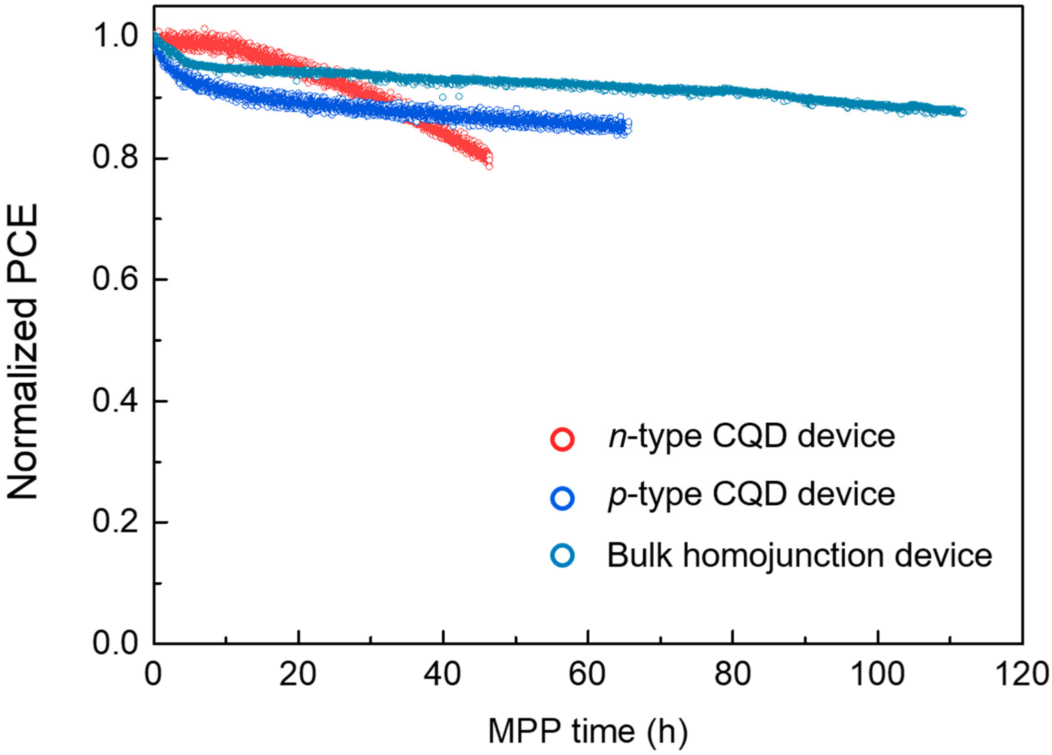

Supplementary Figure 16. Long term device stability with continuous device operation at maximum power point under AM1.5 illuminations in N_2 atmosphere.

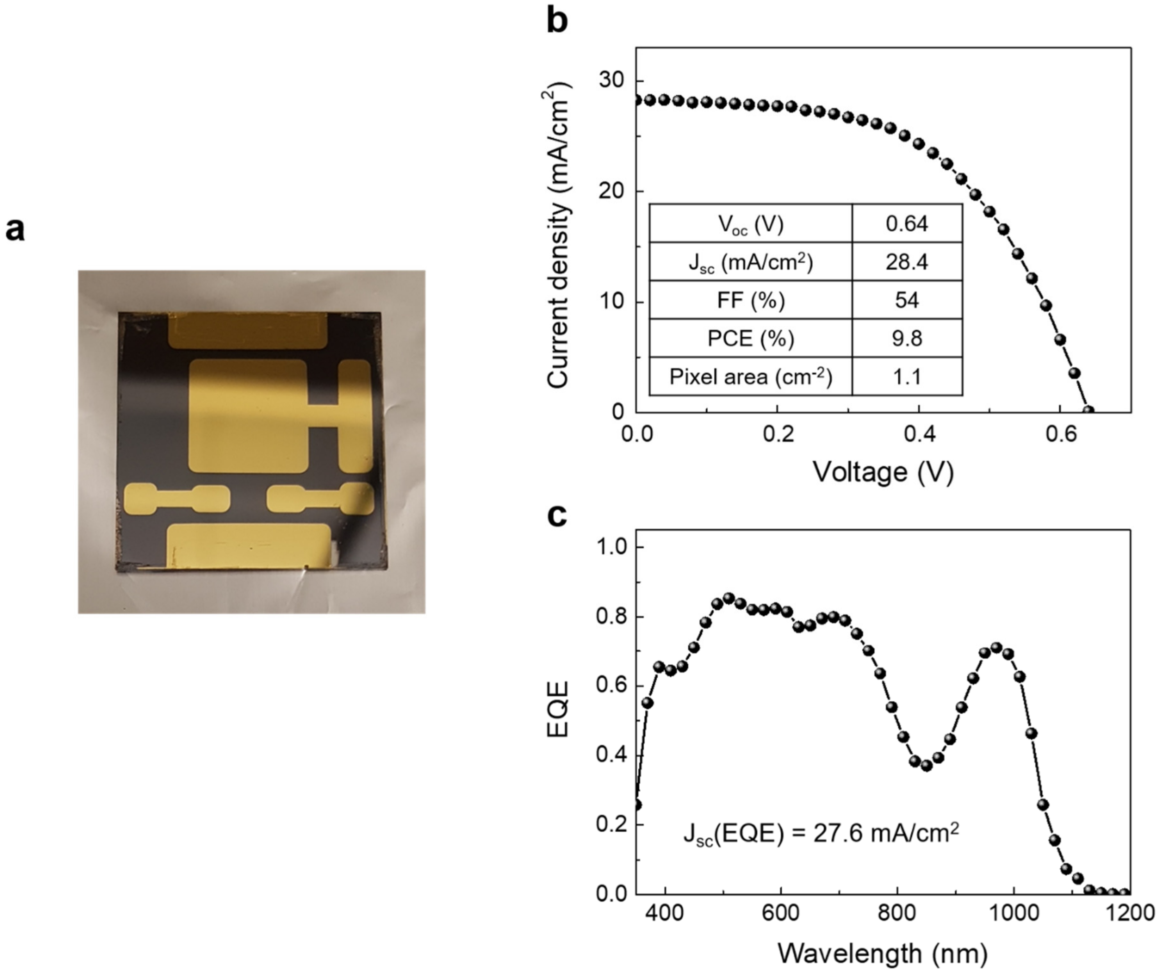

Supplementary Figure 17. a, Photograph image of large-area device fabricated with CQD bulk homojunction solid (active area = 1.1 cm^2; substrate area = 2.5 cm x 2.5 cm). **b,** J-V characteristics and device performance of large-area device. **c,** EQE spectra of large-area device.

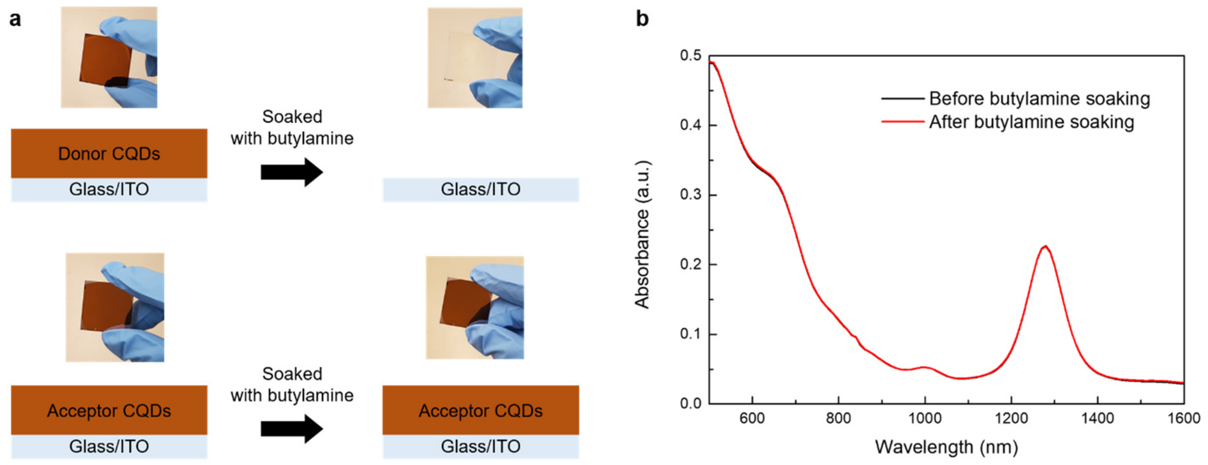

Supplementary Figure 18. a, Schematic and photographs of CQD layers before and after butylamine soaking. While the donor CQD layer is completely redispersed with the addition of butylamine, the acceptor CQD layer does not redisperse nor change. **b,** UV/visible absorbance spectra of the acceptor CQD layer before and after butylamine soaking. The acceptor CQD layer shows the same absorbance spectra before vs. after butylamine soaking.

Supplementary References

1. Speris, M. J. et al. Temperature dependent behaviour of lead sulfide quantum dot solar cells and films. *Energy Environ. Sci.* **9**, 2916-2924 (2016).
