# Supplementary material for: Cascade surface modification of colloidal quantum dot inks enables efficient bulk homojunction photovoltaics
Source: Nat Commun. 2020 Jan 3;11:103. doi: 10.1038/s41467-019-13437-2 (PMC6941986; doi:10.1038/s41467-019-13437-2)
Supplement: Supplementary file 2 — Solar Cells Reporting Summary [file 41467_2019_13437_MOESM2_ESM.pdf]

nature research

Corresponding author(s): Edward. H. Sargent

Solar Cells Reporting Summary

Nature Research wishes to improve the reproducibility of the work that we publish. This form is intended for publication with all accepted papers reporting the characterization of photovoltaic devices and provides structure for consistency and transparency in reporting. Some list items might not apply to an individual manuscript, but all fields must be completed for clarity.

For further information on Nature Research policies, including our data availability policy, see Authors & Referees.

Experimental design

Please check: are the following details reported in the manuscript?

1. Dimensions

Item	Yes/No	Details
Area of the tested solar cells	☒ Yes ☐ No	Described in Methods (Solar cell measurement). The area of certified device was approximately 0.049 cm^2 (Supplementary Figure 14)
Method used to determine the device area	☒ Yes ☐ No	Described in Methods (Solar cell measurement). Optical aperture is used to define the active area.

2. Current-voltage characterization

Item	Yes/No	Details
Current density-voltage (J-V) plots in both forward and backward direction	☒ Yes ☐ No	Newport certificate is shown in Supplementary Figure 14.
Voltage scan conditions<br>*For instance: scan direction, speed, dwell times*	☒ Yes ☐ No	Described in Methods (Solar cell measurement).
Test environment<br>*For instance: characterization temperature, in air or in glove box*	☒ Yes ☐ No	Newport certificate tested in N2. Temperature 25C.
Protocol for preconditioning of the device before its characterization	☒ Yes ☐ No	Described in Methods (Solar cell measurement).
Stability of the J-V characteristic<br>*Verified with time evolution of the maximum power point or with the photocurrent at maximum power point; see ref. 7 for details.*	☒ Yes ☐ No	Long-term stability at the maximum power point is shown in Supplementary Figure 16.

3. Hysteresis or any other unusual behaviour

Item	Yes/No	Details
Description of the unusual behaviour observed during the characterization	☐ Yes ☒ No	No hysteresis was observed for all CQD PV devices, including both the control devices and bulk homojunction devices. The hysteresis-free behavior in CQD PVs is also widely-accepted in literatures.
Related experimental data	☒ Yes ☐ No	Newport certificate is shown in Supplementary Figure 14.

4. Efficiency

Item	Yes/No	Details
External quantum efficiency (EQE) or incident photons to current efficiency (IPCE)	☒ Yes ☐ No	EQE data is described in Figure 5e.
A comparison between the integrated response under the standard reference spectrum and the response measure under the simulator	☒ Yes ☐ No	Described in the manuscript (Figure 5d and 5e)
For tandem solar cells, the bias illumination and bias voltage used for each subcell	☐ Yes ☒ No	Not applicable. No tandem devices were involved in this work.

5. Calibration

Item	Yes/No	Details
Light source and reference cell or sensor used for the characterization	☒ Yes ☐ No	Described in Methods (Solar cell measurement).
Confirmation that the reference cell was calibrated and certified	☒ Yes ☐ No	Described in Methods (Solar cell measurement).

Calculation of spectral mismatch between the reference cell and the devices under test

☒ Yes ☐ No

Described in Methods (Solar cell measurement).

6. Mask/aperture

Size of the mask/aperture used during testing

☒ Yes ☐ No

Described in Methods (Solar cell measurement).

Variation of the measured short-circuit current density with the mask/aperture area

☒ Yes ☐ No

See Methods (Solar cell measurement) and Supplementary Figure 14.
Tested with aperture between solar source and device.

7. Performance certification

Identity of the independent certification laboratory that confirmed the photovoltaic performance

☒ Yes ☐ No

Newport certificate is shown in Supplementary Figure 14.

A copy of any certificate(s)
Provide in Supplementary Information

☒ Yes ☐ No

Newport certificate is shown in Supplementary Figure 14.

8. Statistics

Number of solar cells tested

☒ Yes ☐ No

Total 160 devices were used for histograms described in Supplementary Figure 15.

Statistical analysis of the device performance

☒ Yes ☐ No

Histograms of efficiency are shown in Supplementary Figure 15.

9. Long-term stability analysis

Type of analysis, bias conditions and environmental conditions
For instance: illumination type, temperature, atmosphere humidity, encapsulation method, preconditioning temperature

☒ Yes ☐ No

Long-term stability at the maximum power point is shown in Supplementary Figure 16.
